# Comparing an In-Person and Online Continuing Education Intervention to Improve Professional Decision-Making: A Mixed Methods Study

**DOI:** 10.1177/10497315231185534

**Published:** 2023-06-29

**Authors:** Cheryl Regehr, Arija Birze, Michael Palmer, Karen Sewell, Jane Paterson, Dale Kuehl, Barbara Fallon

**Affiliations:** 1 Factor-Inwentash Faculty of Social Work, 7938 University of Toronto, Toronto, ON, Canada; 2 649424 Institute for Better Health, Trillium Health Partners, Mississauga, ON, Canada; 3 School of Social Work, 6339 Carlton University, Ottawa, ON, Canada; 4 7978 Centre for Addiction and Mental Health, Toronto, ON, Canada

**Keywords:** continuing education, decision-making, online education, simulation

## Abstract

**Purpose:** This paper compares two iterations (in-person and online) of a multi-stage continuing education program for improving high-risk decision-making among mental health workers. **Methods:** The mixed-methods study analyzed the following: (1) physiological and psychological arousal during simulated patient interviews; (2) physiological and psychological arousal recorded during real-time decision-making over four months; and (3) thoughts on the process and outcomes of the intervention raised in reflective interviews. **Findings:** Quantitatively, there were no statistical differences in stress measures between in-person and online simulated interviews or decision-making logs, suggesting they were effective in eliciting reactions commonly found in challenging clinical situations. Qualitatively, participants in both iterations indicated that the intervention caused them to reflect on practice, consider a wider range of factors related to the decisions, and enact approaches to improve decision-making. **Conclusions:** A carefully constructed online continuing education experience can result in outcomes for experienced social workers that are equivalent to an in-person iteration.

Social work scholars and licensing bodies alike assert the importance of continuing professional development, or lifelong learning, as a means to continue providing high quality, evidence-based practices in a rapidly changing environment (BASW, 2012; CASW, 2015; Kurzman, 2016; NASW, 2003). As social workers find themselves at the “intersection of innovation, expanding knowledge, and external scrutiny” (Nissen et al., 2014, p. 385), continuing professional development can serve the needs of the professional, the profession, and the public. However, as Sollars and Xenakis (2021) reflect, social workers and other professionals in health care “practice in fast-paced, demanding work environments with complex cases, a situation not conducive to reflecting meaningfully on their work or to acquiring new skills” (p. 162). These problems are exacerbated when social workers find themselves outside of larger urban centers and consequently have limited access to in-person educational opportunities (Hudson et al., 2021).

Thyer and Pignotti (2016) point to the absence of standards that ensure that social work continuing education programs are evidence-based, and not reliant on “pseudoscience”. Others suggest that continuing education programs suffer from quality control issues, do not lead to transferable skills, and often result in a sense of frustration and disappointment (Gianino et al., 2016). To this end, Nissen et al. (2014) suggest that educators need to be “deliberate and intentional about building updated lifelong learning models into social work practice frameworks and educational structures” (italics in original) (p. 386). We, the authors of this paper, interpret this to have two components: (1) ensuring that the content of continuing education programs is based on best available evidence, and (2) evaluating the ability to continuing education programs to promote the improvement of social work services.

This paper reports on mixed-methods research evaluating and comparing two pilot iterations of a continuing educational intervention for social workers and cognate mental health professionals at the Centre for Addiction and Mental Health, a teaching hospital of the University of Toronto and Canada's top-ranked mental health research hospital. The intervention was aimed at improving professional decision-making in situations of risk and uncertainty. The design was based on extensive research and reviews of the evidence regarding factors influencing professional decision-making and means for improving decision-making (Regehr, 2018). It combined (1) a masterclass series on decision-making science; (2) practice-based self-monitoring with the aid of wearable technology; (3) decision-making logs intended to provoke reflection immediately following a meaningful event, and (4) individual opportunities to engage in guided reflection on action following practice simulations. While we have previously reported on outcomes of an in-person iteration of the pilot program (Regehr, Bogo et al., In press; Regehr, Paterson et al., 2021, 2022), the transition of the intervention to an online delivery format as a result of the COVID pandemic provided an opportunity to redesign the intervention in an online format, and subsequently compare the virtual modification with face-to-face delivery of the first pilot iteration.

## Online Education

Distance learning and more recently online education have a long tradition in universities more generally, and particularly in social work, as programs have sought to meet the needs of community-bound learners. With the onset of the COVID pandemic, university programs across the world moved to online delivery in what might be understood as *online by necessity* (Barton et al., 2022; Regehr & McCahan, 2020). Having had the opportunity to experience the benefits and drawbacks of online education models, universities are now considering which programs and students might best be served through online delivery, which approaches to online education would be most effective, and in which circumstances. This can be thought of as *online by design.* Rigorous research on this issue is relatively sparse but is emerging with mixed findings.

Ebner and Gegenfurtner (2019) conducted a meta-analysis of research conducted before COVID, comparing webinars (synchronous online classes), asynchronous online instruction, and face-to-face instruction in two dimensions: learning and student satisfaction. In the end, only five studies were included in the analysis, demonstrating the relative dearth of rigorous comparative research in this area. Results demonstrated that synchronous online education was more effective in promoting learning than face-to-face instruction, but that satisfaction was greatest in face-to-face, followed by webinars, and finally asynchronous education (Ebner & Gegenfurtner, 2019). Castro and Tumibay (2021) conducted a review that included a comparison of student interactions in face-to-face and online education, similarly finding very few studies comparing the efficacy of these different types of educational experiences. The review determined that group contact was more likely to continue between meetings of the class among online cohorts, that students experienced less anxiety, and that feedback on student work was more extensive, detailed and focused in that it often included written feedback from classmates as well as instructors (Castro & Tumibay, 2021). In a scoping review of online social work education, Afrouz and Crisp (2021) concluded that while satisfaction remained high for online students, communication and engagement presented a challenge when compared to face-to-face instruction.

Earlier research comparing two sections of a course taught by the same instructor demonstrated that students overwhelmingly viewed both types of deliveries to be equally effective and that there were no significant differences in grades (Neuhauser, 2002). In comparing students in a “fully interactive multimedia environment” (p. 49) with those in a traditional classroom, Zhang (2005) found those in the e-learning section achieved higher levels of performance and reported higher levels of satisfaction. More recently, Usher et al. (2021) conducted a study comparing 103 engineering students in a face-to-face course with 108 students in an asynchronous online course, on the level of innovation demonstrated in course assignments. While students in the two modes of delivery reported similar levels of innovation on self-report measures, those in the face-to-face were rated as having higher levels of innovation by independent raters (Usher et al., 2021). Thus, given the limitations created during COVID and changes in the workplace in the post-COVID time, and the challenges in accessing evidence-based continuing education for social workers, there is reason to believe that an online adaptation of a continuing education masterclass designed for classroom-based learning could be as effective, or potentially more effective, in engaging social workers and allied health professionals, but also that some benefits found in in-person instruction may be lost in the online environment.

## Clinical Simulations in Research and Education

Simulation in the form of role play has a long tradition in social work education (Hargreaves & Hadlow, 1997), but has recently become an increasingly sophisticated and standardized method of clinical teaching and performance assessment (Regehr & Birze, 2021). Simulation employs actors (typically in face-to-face encounters) to present realistic client scenarios, allowing for the demonstration and assessment of competence. Originally designed for medical education (Harden & Gleeson, 1979; van der Vleuten & Swanson, 1990), and subsequently adapted for a range of professions (Austin et al., 2003; Parkin & Collinson, 2019), Objective Structured Clinical Examination (OSCE) is a simulation approach that has been validated through extensive empirical research (Hodges, 2006). Bogo and colleagues advanced simulation as an educational tool in social work and a means to assess clinical competence through developing and validating rating scales (Bogo, 2010; Bogo et al., 2012; Kourgiantakis et al., 2020). Although it has been noted that the use in continuing education of social work practitioners is almost non-existent (Kourgiantakis et al., 2020; Sollars & Xenakis, 2021), one study using OSCE-type simulations in this respect has demonstrated that they can be qualitatively experienced as real-life and that physiological and psychological measures demonstrate that participants react to scenarios in ways consistent with actual workplace encounters (Regehr & Birze, 2021).

In general, research conducted using standardized patients has focused on their ability to simulate real practice situations in face-to-face encounters. A notable exception is that of Fang et al. (2013) who modified the OSCE approach for use in cyber-counseling. The onset of COVID threatened the use of the OSCE in teaching and assessment of health professionals and forced programs to develop online approaches for which high levels of satisfaction have been reported by students and faculty alike (Bay et al., 2021; Hannan et al., 2021; Hytönen et al., 2021; Mak et al., 2022; Ryan et al., 2020).

Earlier studies have suggested that virtual simulations (more broadly defined) can contribute to student self-efficacy, as well as the acquisition of social work knowledge, skills, and values (Occhiuto et al., 2022; Reinsmith-Jones et al., 2015; Washburn et al., 2020). Asakura et al. (2020), however, raise a note of caution when reviewing the use of AI generated simulations, especially with respect to the emotional and relational aspects of social work practice. Occhiuto et al. (2022) conducted qualitative research examining student perceptions of learning using online simulated client interviews with standardized patients in response to COVID restrictions. They report that student feedback was highly positive, noting that the simulations realistically reflected the online counseling environment, and did so in a way that felt “safer” and less anxiety-provoking. Notably missing, however, are other measures to assess the ability of online simulated interviews to replicate practice experiences and learning outcomes.

## The Current Study

Social workers and other professionals providing mental health services are regularly required to make high stakes decisions in contested areas characterized by conflicting societal demands. For example, they must consider the rights and freedoms of individual clients to determine the nature of treatment they will receive, with concerns that individuals may commit suicide or harm others. At times, despite best efforts, intentions, and expertise, these decisions result in tragic outcomes. This continuing education project piloted a new approach to improving decision-making through explicit attention to biological, emotional, cognitive, and environmental influences on decision-making during real-world practice experiences, and opportunities for self-reflection following simulated assessment. For the purpose of comparing the in-person and online delivery methods of the program, the current paper analyzes the data collected and used as part of the intervention.

**Figure 1.**
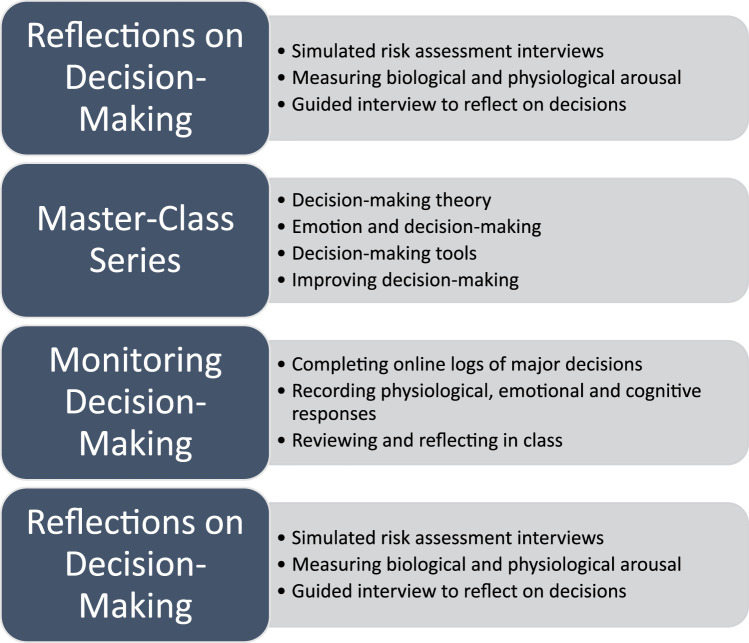
Stages of the intervention.

The intervention comprised a multi-stage process that involved: (1) an OSCE style interview to prompt and capture reflections on decision-making in a simulated practice situation; (2) educating individuals about decision-making processes through a masterclass series that comprised four half-day sessions spaced one month apart; (3) self-monitoring of decision-making during real-world practice aided by wearable technology; (4) post-intervention reflections in a simulated practice situation. The intervention spanned a 4-month period of time from August to December 2019 for the in-person intervention, and from September to December 2022 for the online intervention. A more complete description of the various components of the intervention is found below and is depicted graphically in Figure 1.

### Pre-Intervention Simulated Interviews and Reflections on Decision-Making

The process began with a structured interview and reflection process for each participant using the OSCE approach. The intervention used two realistic and previously validated client scenarios to assess suicide risk (Regehr et al., 2015). Participants completed a 15-min interview with a standardized patient depicting the client. In the in-person iteration, the interviews were conducted face-to-face in an office at the organization in which participants were employed. In the online iteration, participants were in an office at the organization in which they were employed but remotely interacted with the standardized clients via the Zoom videoconferencing platform. At the end of each scenario, participants were asked to state their clinical impression regarding the level of risk the client presented and appropriate case disposition. In addition, participants completed a standardized protocol currently required in their practice setting for suicide risk (Bisconer & Gross, 2007; Cochrane-Brink et al., 2000).

Simulated interviews with standardized patients were video recorded, and videos were played back to participants through a guided interview process, during which they examined and explicated their decision-making process, including cognitive, somatic, and affective influences. This element of the intervention was performed face-to-face with a research assistant in both the online and in-person iterations.

### Masterclass Series on Improving Decision-Making

After completing the interviews, a masterclass series involved four three-hour sessions spaced four weeks apart from one another, developed and led by the first author of this report. Each session included didactic presentation of evidence-based material on decision-making processes and factors affecting decision-making, and robust discussions regarding how the theory and scientific data applied to individual practice, as well as discussions about the decision-making logs that individuals maintained between sessions. The four sessions were as follows: (1) *The Art and Science of Professional Decision-Making* (decision-making theories, the nature of expertise, and the role of expertise in high-risk decision-making); (2) *Emotion and Decision-Making* (impact of emotions and trauma on professional performance and decision-making); (3) *Decision-Making Tools* (strengths and limitations of standardized tools, and the use of clinical overrides); (4) *Improving Decision-Making* (coding decision-making logs in support of collectively designing personalized fast and frugal decision trees). More details on the content of masterclasses can be found in Regehr et al., (2021).

Masterclasses for the in-person iteration were held at the health care organization in which the professionals worked; a light breakfast was provided. Research assistants took notes on participation and key discussion points. Classes were recorded for those unable to attend. Masterclasses for the online iteration were conducted synchronously using Teams for all those able to attend. High-quality pre-recorded versions of the lectures were also available through the course learning management system (a Canvas system locally titled Quercus) for those unable to attend, or those wishing to review material.

### Monitoring Decision-Making

Participants in both cohorts were outfitted with a Garmin vivosmart 4, wearable technology wrist-worn device for use throughout the 12-week duration of the masterclass series. They were instructed on how to use the Garmin for monitoring their heart rate and allowed to keep it after the intervention was completed. Between masterclass sessions, participants continued to wear the Garmin technology, monitored their decision-making processes, and completed online logs when they encountered major decisions. The information contained in the logs included a description of the situation and decision-made, explication of physical and emotional responses and cognitive processes, and heart rate data.

At the outset, it was expected that decisions recorded on logs would occur in client encounters and reflect clinical decisions. However, in keeping with our design-based research method, it became clear in the in-person iteration that stressful decisions often involved staff or other team members, and as a consequence, the definition of decisions was expanded to include these situations. Thus, these types of entries were encouraged from the onset of the online iteration of the intervention.

### Post-Intervention Reflections on Decision-Making

In addition to the reflections on decision-making that occurred during the masterclass series of sessions, approximately one month after the final masterclass, participants completed a second OSCE with the alternate scenario to the one they encountered in the initial assessment. Participants again reviewed their decision-making and reflected on the impact this process had on their decision-making. The breakdown of face-to-face and online interactions matched that of the structured reflections that took place prior to the masterclass series.

## Method

In keeping with the exploratory nature of this stage in our research program, a multi-method design-based research (DBR) framework was utilized. This model that originally arose in the field of curricular design (Brown, 1992) focuses on refining and testing theory while at the same time improving practice through a continuous cycle of design, evaluation, and redesign (Dolmans & Tigelaar, 2012). In this way, design-research is inherently interventionist (Bereiter, 2002; Cobb et al., 2003; Kelly et al., 2014). Theory-based innovations are tested, through mixed-method research (Brown, 1992), “integrating quantitative and qualitative into a cohesive whole” (Bowler & Large, 2008, p. 41) (p. 41), in the context of complex real-world practice where competing variables interact with one another (Collective, 2003; Dolmans & Tigelaar, 2012). As the original concept is subject to cycles of testing in real-world situations in DBR, the model is altered and refined as the research evolves. This requires that community practitioners are full participants in the process (Bereiter, 2002; Cobb et al., 2003), reflecting and offering suggestions for change. In the current study, the iterative improvements focused both on the educational intervention aimed at improving decision-making and on the individual practice of participants.

This project was supported by the Social Sciences and Humanities Research Council of Canada and approved by the Human Subjects Ethics Review Panel of the University of Toronto. All participants provided informed consent.

### Participants

In both the in-person and online iterations, we recruited professional staff employed at the Centre for Addiction and Mental Health. Given the interprofessional model of care in the organization, individuals in allied health professions were invited to participate in the study alongside social workers through a flyer sent via the organization's listserv, asking interested professionals to connect directly with the research study coordinators. Professionals from these groups are all involved in making similar decisions regarding risk assessment; they also learn together in educational sessions focusing on suicide risk assessment.

Participation in the in-person versus the online version was not by random assignment. The first iteration of the pilot was in-person, but we were unable to replicate the in-person as a result of COVID-19 restrictions and therefore moved to online design. However, for the simulated interviews, participants in each iteration were randomly assigned to one of two previously tested (Regehr et al., 2015) scenarios for the pre-intervention simulation and the alternative scenario for the post-intervention simulation.

Thirteen individuals voluntarily engaged in the in-person intervention, 11 women and 2 men. The mean age was 38 with an age range of 25–50. Seven participants identified themselves as White and six as members of other racialized groups (sample size is too small to specify). Participants had worked an average of 10.3 years (range 1.5–23) in the professions of social work (eight), nursing (four), and occupational therapy (one).

Ten individuals voluntarily engaged in the online intervention, nine women and one man. The mean age was 44 with an age range of 25–59. Six participants identified themselves as White and four as members of other racialized groups. Participants had worked an average of 16.4 years (range 1–36) in the professions of social work (seven), nursing (two), and occupational therapy (one).

### Data Collection

Data were collected for multiple purposes: (1) to inform the participants as part of the intervention, (2) to inform the ongoing development of the intervention, and (3) to analyze the impact of the intervention. For this study, we have analyzed the measures to compare the in-person and online delivery methods of the intervention. The research coordinators completed training ahead of the data collection for both phases of the intervention, engaging in experiential activities to ensure accurate use of the technology employed for the study, and administration of measures. The research team then trained the OSCE actors, completing mock collection sessions and engaging in consensus rating discussions.

### Quantitative Measures

#### Pre and Post-Intervention Simulated Interviews

Acute psychological stress during simulated interviews was assessed using the state form of the State-Trait Anxiety Inventory (STAI) (Spielberger, 1983). It consists of 20 statements to which respondents indicate their level of agreement on a 4-point scale regarding how they feel at the given moment. The internal consistency of the STAI-S anxiety scale is high, with alpha coefficients above .85.

Continuous heart rate variability (HRV) was recorded with a FirstBeat BodyGuard 2 HRV monitor, a small and comfortable device affixed to the chest and side with two electrode patches (Parak & Korhonen, 2013) that provided data output for later evaluation.

#### Masterclasses

Data on attendance were collected by research assistants for the face-to-face iteration. Data on attendance and online engagements were collected through the online learning system for the online version.

#### Decision-Making Logs

Resting heart rate data were entered by participants based on their Garmin readings before the stressful situation and maximum heart rate during the stressful decision-making encounter. The decision-making logs prepared by each participant were collected and counted.

### Qualitative Measures

#### Reflective Discussions on Simulated Interviews

Participant reflections after the simulated interviews were elicited through a semi-structured interview, and were audio-recorded and transcribed verbatim for analysis.

#### Masterclasses

Research assistants took notes on discussions during masterclasses for analysis.

#### Decision-Making Logs

Online logs of decision-making contained participant noted reflections on the nature of the stressful encounter, key moments in the decision-making process, physiological responses, emotional responses, and the impacts of physiological and emotional arousal on decision-making.

### Analyses

#### Quantitative

Heart rate recorded on FirstBeat BodyGuard 2 HRV monitors during the simulated interviews was averaged during a five-min segment at six points during each of the pre and post-intervention OSCE interviews: baseline while completing the forms, at the beginning of the OSCE, at the mid-point of the OSCE, at the end of OSCE, and at five and 20 min post-OSCE. Mean differences in HR between online and in-person participants were compared using independent samples t-tests. Change in heart rate between the six timepoints was analyzed using a one-way repeated measures ANOVA.

Subjective stress was recorded using the STAI at 5 points during each of the pre and post testing sessions: baseline while completing forms, immediately before the OSCE, immediately after the OSCE, immediately after completing the suicide risk assessment, and after the video reflection interview towards the end of the session. Mean differences in STAI scores between online and in-person participants were compared using independent sample t-tests. Change in STAI scores between the five timepoints was analyzed using a one-way repeated measures ANOVA.

Heart rate recorded by participants in the decision-making logs shortly after the incident was summarized descriptively and compared using independent sample t-tests. Number of decision-making logs and participation in sessions were summarized descriptively.

All statistical analyses were conducted using SPSS Statistics v. 28.0.0.1 (14).

#### Qualitative

Qualitative data drawn from the participant-generated decision-making logs, from notes arising from participant discussions generated in the four monthly masterclasses, and transcriptions of the reflective interviews conducted following the simulated interview one-month post-intervention, were repeatedly read by the Principal Investigator (PI) of the study who coded the data line-by-line using constant comparative analysis to organize the data into focused codes. The PI then engaged with other members of the research team to gain their various perspectives on the evolving codes. These discussions generated an understanding of the connections between the codes and their meaning.

## Results

Given the multi-stage, mixed-methods design of this study, results are separated into quantitative findings (physiological and psychological arousal during simulated patient assessments and recorded in decision-making logs, masterclass participation, and quantity of decision-making logs) and qualitative findings (context factors included in decision-making logs and participants’ thoughts about the process and outcomes of the intervention raised in reflective interviews).

### Quantitative Findings

#### Simulated Patient Assessments

**Figure 2.**
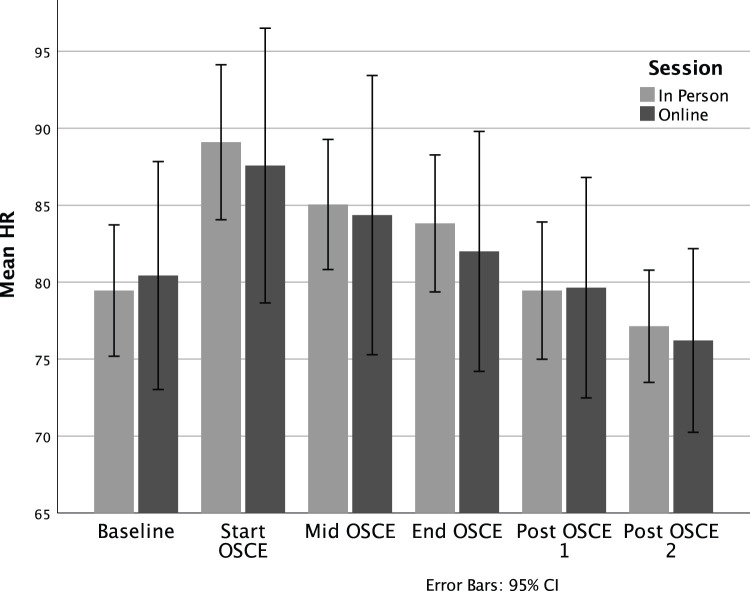
Heart rate during in-person and online simulated patient assessment.

We have previously demonstrated the effectiveness of simulated interviews in creating a stressful decision-making situation that might closely reflect a real-life practice encounter (Regehr, Bogo et al., 2015; Regehr, Shlonsky et al., 2010). One aspect of this is physiological and psychological arousal. As demonstrated in Figure 2, heart rate rose to a high point at the beginning of each OSCE, remained elevated during the OSCE, and then went down at 5-min and 20-min mark after it ended. Of note, independent sample t-tests revealed no statistical difference in heart rate at each timepoint between the two models of delivery, suggesting that the two delivery methods were equally able to produce physiological arousal. Because there were no differences in HR between groups, the samples were then combined to conduct the one-way repeated measures ANOVA, examining HR change over the six timepoints. This revealed a significant effect of time, Wilks’ Lambda = .33, *F*(5, 31) = 12.77, *p* < .001, η^2^ = .67. Follow up comparisons indicated that significant pairwise differences are seen between each timepoint (*p* < .05). That is, there is a significant increase at Time 2 from Time 1, followed by significant decreases from Time 2 to 3, 3 to 4, 4 to 5, and 5 to 6.

**Figure 3.**
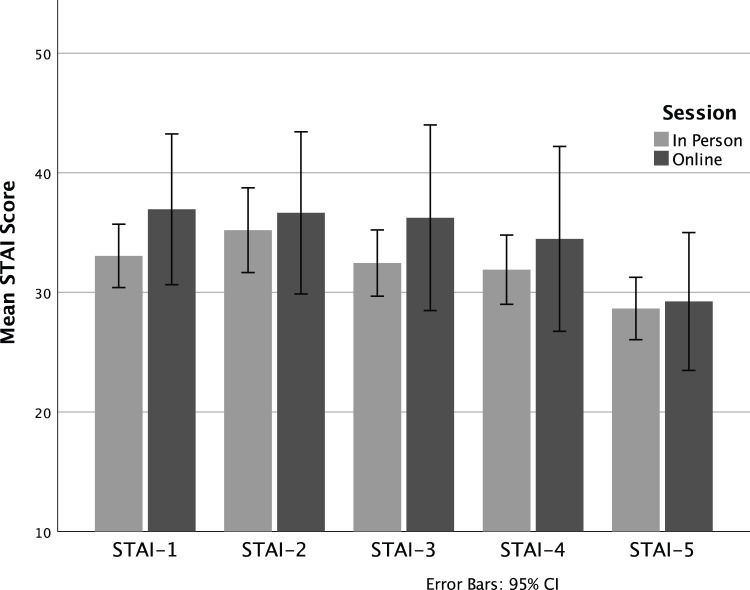
Subjective anxiety during in-person and online simulated patient assessment.

In a similar manner to heart rate, mean STAI scores rose from baseline to a highpoint at the onset of both OSCEs, and then diminished during the recovery period (Figure 3). In reflective discussions in the masterclasses, participants affirmed that they experienced the highest levels of arousal as they were entering a real-life high-risk decision-making situation. Of note and in a similar fashion to HR, there are no statistical differences in STAI scores between the two models of delivery, suggesting that the two delivery methods were equally able to produce psychological arousal. Because there were no differences in STAI scores between groups, the samples were then combined to conduct the one-way repeated measures ANOVA, examining change in subjective stress over the five timepoints. This again revealed a significant effect of time, Wilks’ Lambda = .38, *F* (4, 33) = 13.54, *p* < .001, η^2^ = .62. Follow up comparisons indicated that significant pairwise differences are seen between Time 5 (final resting assessment) and all other timepoints (*p* < .01). That is, while subjective stress remained heightened and relatively stable throughout the session, by the final assessment, participants had returned to lower than baseline levels of subjective stress.

#### Masterclass Participation

Participation in the in-person version of the masterclass began strong but diminished over time as work and life responsibilities interfered. One person was unable to complete the in-person masterclasses due to a change in work assignments; attendance for the first class was 92% (*n* = 11), the second 83% (*n* = 10), and 58% (*n* = 7) for the third and fourth classes.

With respect to the online intervention, two people dropped out part way through the process; attendance in the first class was 70%, the second 60%, the third 40%, and the fourth 50%. One of the participants who attended synchronous classes missed portions of the classes (for instance having a meeting that interfered); this individual also watched the class videos online. Two participants (20%) who attended the synchronous classes sporadically (once or twice) and the remaining four individuals who did not attend the synchronous classes engaged with the course on the LMS, ranging from 26 to 144 interactions online. Thus, while sporadic or non-attendance felt disruptive (at least to the facilitator) from a group process perspective, participants nevertheless found a way to engage with material.

#### Decision-Making Logs

During the in-person version of the intervention, 91 logs with heart rate data were submitted. Change in heart rate from prior to the decision-making situation to during the decision-making was calculated, and the degree of change was divided into three categories. Heart rate change of under 10 beats per minute (bpm) was classified as no change or low change and was recorded in 27 (29.7%) of logs. Moderate change (10–19 bpm) was recorded in 27 (29.7%) of logs. High change of 20 bpm or greater was recorded in 37 (40.0%) of logs. Situations in which a high degree of heart rate change was identified included client challenges, organizational challenges, managing team dynamics, and socio-evaluative situations such as giving a presentation.

For the online version of the intervention, 32 logs were submitted with heart rate data. Heart rate change of under 10 beats per minute (bpm) was classified as no change or low change and was recorded in six (18.8%) of logs. Moderate change (10–19 bpm) was recorded in 10 (31.3%) of logs. High change of 20 bpm or greater was recorded in 16 (50%) of logs. Situations in which a high degree of heart rate change was identified included sharing feedback with a peer and supervising staff, voicing opinions in team meetings, making decisions about clients, and organizational logistics. Interacting with other staff was most frequently cited. Thus, online participants were less likely to complete decision-making logs, but when they did, focused on situations that resulted in higher levels of physiological arousal.

Of note and in a similar fashion to HR and STAI, there are no statistical differences in HR change scores between the two models of delivery, suggesting that HR change during decision-making events throughout the intervention was similarly producing psychological arousal regardless of the model of intervention delivery.

### Qualitative Findings

#### Simulated Patient Assessments

We have previously reported that participants in in-person versions of simulated patient interviews identify that standardized patients portray very life-like patients (Regehr, Bogo et al., 2015; Regehr, Paterson et al., 2021). Similarly, individuals who engaged with online simulated patients indicated that the “simulation was very realistic”. One participant indicated that almost all of her client work is in-person, and yet “I felt like she was right here in front of me, it felt like we were in person.” Another stated “bits and pieces of her could have been in a lot of clients I’ve seen” and “she's my daughter's age, so I do think about a young person that age and feeling that hopeless and…wanting to make sure she stays safe.”

#### Decision-Making Logs

In both in-person and online interventions, emotions associated with elevated heart rate included feelings of dread or “a bad gut feeling”, anxiety or feeling “jumpy”, fear for the safety of clients and fear regarding how colleagues may view the participant, and anger at colleagues. Physical responses included an awareness of having a racing heart, sweaty palms and flushing, muscle tension, shallow breathing, and experiencing “butterflies” or “nervous stomach”.

#### Reflections on the Process and Outcome of the Continuing Education Intervention

Participants in both modes of delivery reflected on what they learned as a result of engaging in the intervention.

I learned all the pieces that go into why I’m making decisions that I am making and that they’re not just random. And I trust them more and understand them more…there's a lot more that I am bringing to a decision than just my gut and a piece of paper and a tool. That I’ve amassed continued experience and knowledge and that's all feeding my decision-making. (Online)

What I’ve learned is that there are key moments. It seems obvious, but it was not something I was ever aware of because my suicide risk assessments were always kind of a blur…So there are these key moments in which you make your decisions and it's important to hone in on those and explore further. (In-person)

I learned just be conscious of what I am asking, and why I am asking the questions that I am, what is that really leading me to?…[I consider] if I’m being client-centered with somebody, or the decision is driven from some other means. Like if I am just really stressed out in the moment and that's leading me to make a decision for myself, and not for the client. (Online)

I definitely feel more confident in my decision-making process as a result of this participation. I think I will be applying what I learned in my daily practice. (Online)

An overriding theme in both the in-person and online versions of the intervention was the importance of collegial engagement. One participant noted:

[J]ust hearing that other social workers also go through this, and it's really difficult, and how anxiety provoking it is…I think makes it so much better…That time I joined in [the synchronous class], just seeing people's faces…it just reassures me that I’m not the only one quivering in the back, hoping everything is going to be ok. (Online)

Similarly, a participant in the in-person stated:

It's been helpful to hear of the decisions others need to make. I don’t feel that we’re always able to discuss our own vulnerabilities, but here they were able to be discussed and that's been helpful. Broken down a bit of isolation. (In-person)

Some of those in the online version appreciated the flexibility “I liked that there were recordings, so someone like myself who was unable to attend, it helped me a lot. I can watch it any time for asynchronous.” “I liked the classes, I liked attending them, I enjoyed by the [synchronous] and watching them.” Others found the technology difficult “the online thing was very frustrating”.

Finally, one person reflected on the value of being part of a study evaluating an intervention to improve decision-making:

I think the allied health team often feels we get left out of research. I mean, event just with the pandemic you hear it, health care workers, everyone thinks of doctors, nurses, maybe PSWs. But I’ve come to work every day during the pandemic. And so have my behavioural therapy colleagues, my OT colleagues, my pharmacy colleagues. And so, we often feel like we get left out. So the research, it was just really validating. (Online)

## Discussion and Applications to Practice

Continuing professional development is critical to ensuring high-quality services are provided by social workers and other allied health professionals, as they are called upon to exercise judgment and make high-stakes decisions in situations of risk and uncertainty. However, conflicting demands on time and energy, as the volume of those seeking services increases, as the regulatory environment shifts, and as resources are increasingly stretched, mean that time to reflect on practice and acquire new skills is often limited (Sollars & Xenakis, 2021). Thus, social work educators must find new ways to engage social workers in continuing education that directly applies to practice (Nissen et al., 2014), and can be integrated into their busy work schedules. In addition, educators as delivers of continuing professional development and practitioners as consumers are reminded that continuing education offerings must be evidence-based in order to ensure that time is well spent and will lead to enhanced practice (Thyer & Pignotti, 2016).

Previously we have reported on an evaluation of a pilot intervention aimed at improving professional decision-making in situations of risk and uncertainty (Regehr, Bogo et al., In press; Regehr, Paterson et al., 2021). We suggested that the findings of the evaluation were heartening and that the intervention held promise for refinement and replication, arising from a design-based research framework. As the COVID pandemic ensued shortly thereafter, and in-person replication was not possible, we designed and implemented an online version of the intervention, hoping to address some of the challenges related to accessibility and calendaring that were inherent in the in-person model.

Participants in both the in-person and online versions of the intervention were given the opportunity to engage in an intensive four-month continuing education program involving simulations with standardized clients and opportunities to examine their decision-making through watching video recordings of their own interviewing, learning about decision-making science and theory in a masterclass series, focusing on professional decisions through the use of decision-making logs with the benefit of physiological data supplied by wearable technology, and sharing and reflecting on decision-making with colleagues.

One aspect of the intervention was an OSCE style-simulated patient assessment and reflective interview. Our first question therefore was whether an online synchronous interview with a standardized patient was as effective in replicating a clinical encounter as an in-person simulation. To this end, physiological stress as measured by heart rate elevation, and psychological stress as measured by the STAI, demonstrated that both iterations were effective in eliciting symptoms of stress commonly found in stressful work situations. This is consistent with previous laboratory and simulation research (Dolan, 2002; Dunn et al., 2010). In addition, qualitative comments of participants supported the realism of an online simulation, a finding that replicates that of others (Occhiuto et al., 2022).

A second consideration was engagement in the continuing education process through attendance of masterclasses or watching videos of the material, and through maintaining decision-making logs. Attendance in the four, once-monthly masterclasses followed a similar pattern of decreased attendance over the four sessions, despite qualitative feedback that they were useful and engaging. Participants identified work conflicts as the primary cause of non-attendance. However, those in the online version of the intervention continued to engage with the materials by watching the videos located on the learning management system.

With respect to decision-making logs that were aimed at provoking real-time opportunities to reflect on practice, those in the online version were less likely to keep logs. This perhaps speaks to the need for facilitators to reach out more directly to online participants, encouraging them to reflect on a greater range of practice encounters. Nevertheless, decision-making logs from both online and in-person participants clearly identified emotional responses that accompanied increased heart rates and high-stakes decisions (Dane et al., 2012; LeBlanc et al., 2014; Slovic et al., 2004).

Participants in both the online and in-person version indicated that that the intervention caused them to reflect on practice, consider a wider range of factors related to the decisions they were making, and how they then used approaches others have identified as improving decision-making (Croskerry et al., 2013; Graber et al., 2012; Moulton et al., 2010; Saltiel, 2016). These approaches included acknowledging the complexity of the situation and consciously gathering and sifting through information, tuning into and seeking to understand their emotional and physiological arousal, and slowing down the decision-making process. Further, similar to that of other researchers (Occhiuto et al., 2022; Reinsmith-Jones et al., 2015; Washburn et al., 2020), participants identified that by engaging in the continuing education program and through using techniques and knowledge acquired, they felt more confident in their decision-making abilities. These preliminary results suggest that using tools such as increasingly ubiquitous wearable technologies to call attention to intuitive processes has promise for social work as well as other professions providing mental health services.

Finally, those participants in the in-person version and those who attended even one or two of the online synchronous masterclasses note the importance of engaging with colleagues, learning from one another, and seeing that they were not alone in struggling with difficult decisions. Authors in other disciplines have noted the importance of developing a learning community in continuing education, both for creating a space for reflection and motivating ongoing efforts to learn new skills (Farrell et al., 2021; Hsiang-Te Tsuei et al., 2019). In addition, participants noted the support that they felt from the organization, simply by being given the opportunity to engage in a continuing education program that was tailored to their environment.

This study demonstrates the challenges and limitations of a real-world intervention. First, the study began as a small in-person pilot with the intention that subsequent iterations would result in larger numbers of participants to test the model. A global pandemic thwarted these intentions and resulted in a reworking of the model as an online intervention. Nevertheless, the aftermath of COVID-19 in the hospital sector resulted in continuing workforce challenges and we were only able to recruit another small cohort for the second iteration. Thus, the result is a small pilot study with only 13 participants in the in-person and 10 participants in the online, conducted in one organization. Further, random assignment into the two modes of delivery was not possible given the exigencies that existed. The generalizability of findings is therefore limited.

In keeping with design-based research, these two pilot studies provide a foundation on which to build, improving the materials presented in the masterclass series and encouraging all participants to complete a greater number of decision logs. Primary recommendations for change by members included either increasing or decreasing the time between masterclass sessions, reducing the length and increasing the frequency, moving the day of the week masterclasses were held, and increasing prompts and reminders for using a wrist-worn device and completing decision logs.

The rapidly changing environment and high stress environments in which social workers work, simultaneously demands that social workers engage in continuing professional development in order to ensure that their skills and abilities continue to serve our clients, and limits the time and resources available to engage in continuing education. Thus, educators are called upon to develop models for professional development that engage practitioners, are directly applicable to their practice, and are flexible to accommodate to other demands on their time.

This paper compares an online continuing education program aimed at improving professional decision-making in situations of risk and uncertainty with an earlier piloted in-person version. Results are highly promising and demonstrate that a carefully constructed online experience can result in outcomes that are equivalent to an in-person iteration. Further, this continuing education approach provides a model, demonstrating the possibilities for integrating research, practice and education with the end goal of improving social work practice and better serving our clients.
